# Ecology and Evolution of the Human Microbiota: Fire, Farming and Antibiotics

**DOI:** 10.3390/genes6030841

**Published:** 2015-09-08

**Authors:** Michael R. Gillings, Ian T. Paulsen, Sasha G. Tetu

**Affiliations:** 1 Department of Biological Sciences, Macquarie University, Sydney, NSW 2109, Australia; 2 Department of Chemistry and Biomolecular Sciences, Macquarie University, Sydney, NSW 2109, Australia; E-Mails: ian.paulsen@mq.edu.au (I.T.P.); sasha.tetu@mq.edu.au (S.G.T.)

**Keywords:** integron, evolution, mercury, disinfectant, resistance, antibiotic, Anthropocene, dysbiosis

## Abstract

Human activities significantly affect all ecosystems on the planet, including the assemblages that comprise our own microbiota. Over the last five million years, various evolutionary and ecological drivers have altered the composition of the human microbiota, including the use of fire, the invention of agriculture, and the increasing availability of processed foods after the Industrial Revolution. However, no factor has had a faster or more direct effect than antimicrobial agents. Biocides, disinfectants and antibiotics select for individual cells that carry resistance genes, immediately reducing both overall microbial diversity and within-species genetic diversity. Treated individuals may never recover their original diversity, and repeated treatments lead to a series of genetic bottlenecks. The sequential introduction of diverse antimicrobial agents has selected for increasingly complex DNA elements that carry multiple resistance genes, and has fostered their spread through the human microbiota. Practices that interfere with microbial colonization, such as sanitation, Caesarian births and bottle-feeding, exacerbate the effects of antimicrobials, generating species-poor and less resilient microbial assemblages in the developed world. More and more evidence is accumulating that these perturbations to our internal ecosystems lie at the heart of many diseases whose frequency has shown a dramatic increase over the last half century.

## 1. Introduction

Humans have become the most important evolutionary force on the planet [1]. Our activities have significant effects on the climate, atmosphere, hydrosphere, and on global biogeochemistry [2]. These changes are leading to intense rounds of natural selection, adding to the pressures already imposed by extinction, homogenization of species distributions, and human technologies that direct evolution [3,4]. The influence of human activities is pervasive, even extending to the microbial world, where we have impacts on dispersal, population structure, and the contributions of microorganisms to biogeochemical cycles [5,6].

We have significantly influenced the biosphere over the last 10,000 years. Across the same time period we have also influenced the composition, properties and diversity of our internal biosphere, the microorganisms that comprise the human microbiota [7]. These microbial cells outnumber our human cells by ten to one, exhibit diversity between body sites and between individuals, and contain two orders of magnitude more genes than the human genome [8,9].

Perturbations to the composition and functions of the microbiota may have significant effects on health [10]. Understanding and dealing with the negative consequences of such dysbiosis requires an understanding of what a “normal” microbiota should look like [11]. It also requires an understanding of the dynamics of our internal microbial communities. In this regard, our microbiota can be examined in the same way as more conventional ecosystems, by analyzing colonization, competition and selection [12].

There is no doubt that the human microbiota has been subjected to various selection pressures over the last 10,000 years. Selective sweeps probably accompanied changes in diet resulting from the spread of agriculture. Direct selection pressures were undoubtedly imposed by increasing exposure to environmental contaminants after the industrial revolution, and by heavy metals, disinfectants and antimicrobial agents in the modern era. Here we review the ancestral microbiota, and examine changes in diversity and composition wrought by various selective agents, including those most potent agents of selection, the antibiotics. To begin this analysis, we concentrate on the genes encoded by the human microbiota: the human microbiome.

## 2. The History of the Human Microbiome

The term “microbiome” has been used in a number of different ways over the past decade. Here we will refer to the human microbiome strictly as the sum total of the genes carried by micro-organisms commensal with humans. This is to distinguish this concept from the human microbiota, which are those species of micro-organisms commensal with humans [13]. It is also important to distinguish between an individual’s microbiome/microbiota, and those of populations, or those of the whole human species.

Does the microbiome have a “steady state” and has the human microbiome changed over time? While the microbiota of modern, westernized populations differs from individual to individual, the functions encoded by their microbiome are much more constant. In particular, the presence and abundance of genes assigned to conserved orthologous groups (COGs) remain similar [10]. In other words, the core functional microbiome remains relatively constant, despite the species housing these functions varying considerably between individuals.

While the core functions of the microbiome might be well conserved, some elements of the microbiome can confer important advantages, while being a comparatively rare component of the total microbiome. These “accessory” genes are not part of the core genome of any particular species, and may only find temporary residence within the microbiome. Bacteria exchange such accessory genes via the process of lateral gene transfer (LGT), itself mediated by the processes of conjugation, transformation and transduction [14].

The human microbiome interacts with the global pangenome (the sum of all genes in the biosphere) through environmental exposure and LGT. Potentially any gene in the biosphere can find its way into the human microbiome by lateral transfer [15]. There are some routes of LGT that are well trodden. Gene exchange is easier to accomplish between organisms in the same Phylum, those with similar GC content, and those that inhabit similar niches [16,17]. In some cases, there are communities where LGT is common, creating networks of gene exchange [18,19]. High cell density and biofilms improve the efficiency of transfer [20]. This makes the high densities and biofilm communities that comprise the human microbiota a potential hotspot for gene exchange, and accumulation of accessory genes [21,22].

To persist in a population, including within the microbiota, a newly acquired accessory gene usually has to be: (i) stably inherited, by incorporation into a replicon; and (ii) able to confer some advantage to the host cell, such that it is not lost by drift or purifying selection [14]. Consequently, we might envisage the human microbiome as a clearing house, where diverse genes constantly arrive by LGT, and are then eliminated by failure to replicate, or are subjected to fixation or elimination by natural selection.

This must have been the state of the human microbiome during our early evolution. Because diet strongly affects the composition of the microbiota, it also affects the microbiome [13], and the likelihood of successful LGT. For instance, distantly related members of the phylum *Bacteroidetes* can readily share genes for degradation of refractory high molecular weight polysaccharides [23], providing an advantage to human populations that rely on fibrous plant foods [24]. Subsequent changes to diet, such as the increasing consumption of cereals after the adoption of agriculture, would then select for altered components of the microbiota [25,26], and simultaneously select for new accessory genes in the microbiome. Adoption of unique dietary components would also promote selection of appropriate accessory genes. For example, a gene for the enzyme porphyranase appears to have made a successful lateral transfer from a marine *Bacteroides* into *B. plebeius* in the microbiota of the Japanese population [27]. This enzyme helps digest seaweed, a common item in Japanese diet. Another example might be the significant change in oral microbiota following the increased availability of processed foods and refined sugar after the industrial revolution [28].

**Figure 1:**
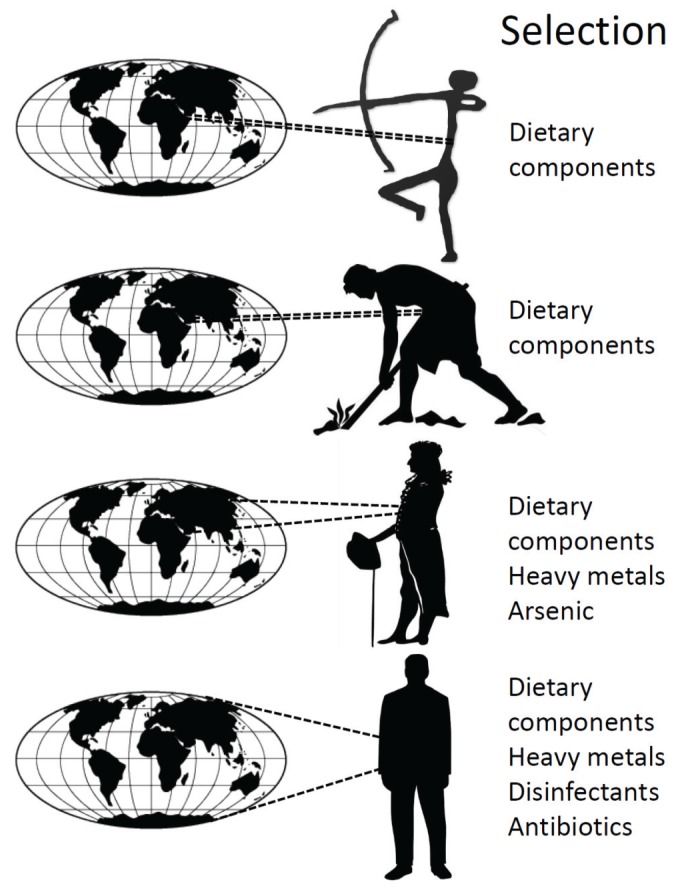
Schematic history of selective forces acting on the human microbiome and microbiota. As human populations moved from hunter-gatherer, to agrarian communities, to the industrial revolution, to modern civilization (vertical dimension), the selective forces on the microbiota and microbiome have changed. Early selection was mainly driven by ability to metabolize dietary components, while more recently, strong selective agents such as heavy metals and antimicrobial agents have become increasingly important. In addition, the potential sources of both microbiota and microbiome components (species and genes) have broadened as mass movement of materials and organisms became a feature of a globalized world.

There are two main differences between the dynamics of ancestral human microbiomes and those of humans living in the developed world. The first of these is that ancient humans and early agrarian populations were exposed to the microbiota and microbiome from a comparatively small geographic area (Figure 1). The diversity of microbiota and microbiome components to which individuals could potentially be exposed increased during the age of exploration, as the movement of humans, plants and animals around the world had an increasing impact on microbial biogeography [6]. In the developed world, an individual has the potential to be exposed to microbiota and microbiome components from anywhere in the world (Figure 1), as demonstrated by the ability of antibiotic resistant enterobacteria to rapidly spread via international travel [29].

The second difference in microbiome dynamics is driven by the mode and strength of selection on microbiome components. In hunter-gatherers and agrarian communities, the microbiota and its component microbiome are subject to selection mainly through diet. This selection is essentially “soft”, in that it arises through competition between individual cells and species in their ability to metabolize food sources. Cells might gain an incremental metabolic advantage by acquiring a new microbiome component via LGT, and thus increase in relative abundance. In contrast, after the industrial revolution, humans have been increasingly exposed to many more agents of “hard” selection (Figure 1). These agents, such as heavy metals, disinfectants, biocides and antibiotics act directly, killing or severely debilitating many cells, while positively selecting those rare cells that carry resistance determinants (serendipitously acquired by LGT). The speed and intensity of this selection has a second consequence, the fixation of particular clonal lineages from within the microbiota. Repeated rounds of selection by different agents can also then result in the sequential assembly of multiple resistance determinants, each with different phylogenetic origins, into complex, mosaic DNA elements [30,31].

## 3. Assembly of Multi-Resistance DNA Elements within the Microbiome

Genes that can confer resistance phenotypes are collectively termed the resistome [32,33,34]. The resistome is a useful organizing concept from a human and medical perspective, but it is not a natural or functional grouping, as it comprises diverse genes whose functions may have simply been co-opted to confer resistance [15,35]. Nevertheless, the resistome concept helps to highlight the point that resistance genes in pathogens often originate from unrelated environmental organisms, and that the resistance genes of medical concern are just a small proportion of the resistome [36,37].

It is important to distinguish between sequences that are assigned to the resistome based on bioinformatic prediction of function, as opposed to genes that confer a demonstrable resistance phenotype. Many resistome elements do not confer resistance in their native context because they are not expressed, or require critical mutations to confer resistance [35,38]. The ancient human microbiota probably did contain diverse elements that could be assigned to the resistome, but these did not confer resistance in the clinical sense [11]. Culture independent sampling of human microbiota reveals a vast reservoir of potential resistance genes. However, the majority of these genes have not been identified in clinical contexts, and are evolutionarily distant from known resistance determinants [39]. In contrast, if aerobic proteobacteria are cultured from fecal samples, over half of the resistome elements are identical to known resistance genes. These resistance determinants are readily exchanged with pathogens, and spread rapidly between individuals [40]. This suggests that proteobacteria from environmental sources might act as a conduit for clinically relevant resistance genes to make their way into the human microbiome and thence into pathogens [14].

Before the Industrial Revolution, it is unlikely that clinically relevant resistance genes were at high frequencies in the human microbiome. Resistome elements would have occasionally found their way into the microbiome via LGT, but in the absence of any selective advantage would be lost by drift or purifying selection. This view is supported by characterization of plasmids collected before the antibiotic era. These plasmids have similar backbones to modern plasmids, but lack resistance genes [41].

**Figure 2:**
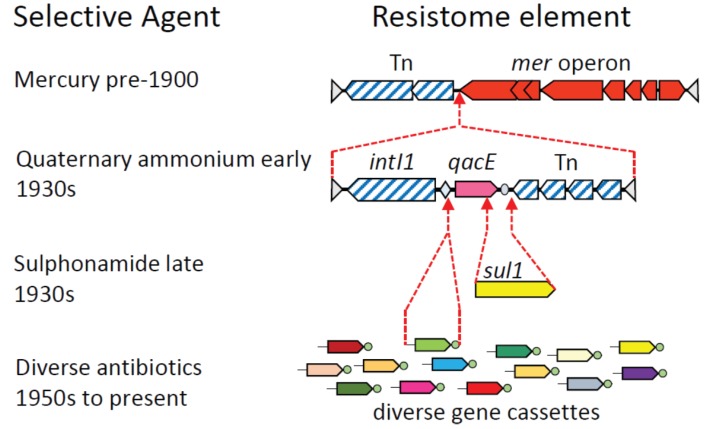
Schematic illustrating the sequential assembly of complex, mosaic DNA elements under the influence of human selection. The proposed history of transposon Tn*21* is used as an example [43,49,50]. Open reading frames conferring resistance phenotypes are represented by solid color; genes for DNA recombination, such as the integron-integrase *intI1* and transposition machinery, have diagonal hatching. The sequential series of selection events that is thought to have fixed each additional resistance gene to form larger and more complex elements are shown. These resistance determinants include: a *mer* operon (mercury resistance); *qacE* (resistance to quaternary ammonium compounds); *sul1* (resistance to sulphonamides); and integron gene cassettes (resistance to diverse antibiotics). Only one of the many acquisitions of different integron gene cassettes is shown. For details of integron diversity and activity see [51].

The first rounds of hard selection to retain resistome elements in the microbiome may have occurred some 120–180 years ago, when there was a significant shift in abundance and diversity of mercury resistance genes in lake sediments. This shift was presumably driven by increased mercury pollution as a consequence of industrialization [42]. Certainly by the late 1800s, both mercury and arsenic were being used prophylactically, and this would have increased the abundance of relevant resistance genes in the microbiome, in particular transposons carrying *mer* operons [43] (Figure 2).

The subsequent development and use of diverse antimicrobial agents during the 20th Century introduced sequential selection pressures, each of which favored cells that happened to have recently acquired an appropriate resistance gene. There is a strong stochastic nature to these events, because two criteria must be met: the acquisition of a relevant element of the resistome; and the coincident application of a selective agent. Consequently, there would be a strong founder effect. Once a particular resistance gene was driven to high abundance by selection, there would be less chance that a second gene could achieve similar success, even if it conferred the same phenotype. This may explain why only certain genes from within the resistome have become clinically important [32,37,44,45].

Each selection event helped raise the abundance and distribution of resistome elements and their mobile DNA vectors such as transposons and plasmids. Recruitment of new resistome elements into existing transposons or plasmids increased the potential for co-selection based on the possession of multiple resistance determinants [46,47,48]. In particular, the acquisition of gene accumulation platforms such as the class 1 integron [49], increased the flexibility and rate at which new resistance determinants could be added to existing multi-resistance elements.

Each extant plasmid, integrative conjugative element or compound transposon has its own unique evolutionary history, which, given the promiscuous nature of lateral gene transfer, might never be precisely reconstructed. Nevertheless, a general illustration of the processes involved can be given by outlining the most likely explanation for the origins and subsequent evolution of the transposons and clinical class 1 integrons that have played such a central role in the dissemination of antibiotic resistance (Figure 2).

Assembly of composite, mosaic resistance elements probably began with exposure to mercury in the 19th and early 20th Century. Environmental or prophylactic mercury exposure may have fixed a *mer* operon and attendant transposon in the human microbiome. The introduction of quaternary ammonium compounds as disinfectants [52] then provided a second selective force. A class 1 integron that carried a *qacE* gene cassette was captured from a betaproteobacterial chromosome by a Tn*402* transposon [49,51], and this hybrid element was then inserted into a *mer* transposon [43]. The resultant mosaic transposon conferred resistance to mercury, and also to disinfectants via the *qacE* integron gene cassette (Figure 2). Both the *mer* operon and the class 1 integron were probably derived from environmental microorganisms, while the Tn*402* transposon might have already been resident in the human microbiome [49]. The fusion of these three elements resulted in the compound transposon Tn*21*, whose subsequent derivatives have become widely distributed on different plasmids in the human microbiome [43,53].

Introduction of sulphonamides, the first true antibiotic, in the late 1930s [54] provided the selective pressure to fix an insertion of the *sul1* gene into the class 1 integron, deleting the terminal portion of the *qacE* gene cassette [50]. Introduction of further antibiotics over the next 60 years selected, at each time point, for integron variants that had acquired the appropriate resistance genes (Figure 2). Over this time period, class 1 integrons captured over 130 distinct antibiotic resistance gene cassettes [55]. The transposons in which the class 1 integrons are embedded have themselves moved between different plasmids, and have also moved by LGT to diverse species, such that class 1 integrons are found in 40%–70% of Gram-negative clinical pathogens [56,57]. They are also commonly carried in the microbiota of humans, their livestock, and domestic animals [14,58], and have become a permanent feature of the microbiome.

There are a number of points to be made here about the influence of antimicrobials on the human microbiome. Antimicrobial compounds provide the motive force to fix appropriate resistance genes when these arrive in the microbiome via LGT. Each additional antimicrobial treatment promotes insertion of more resistance determinants into compound elements of growing complexity. The individual components of these mosaic, multi-resistance elements are derived from diverse, unrelated organisms [31]. The fusion of genes with such disparate origins over such a short time frame would not have occurred but for the sequential application of different selective agents in the modern era. In this sense, the multi-resistance elements now circulating in the human microbiome are xenogenetic, that is, foreign to the natural world, and fixing only through human activity [15,59].

Further, the linkage of diverse resistance genes on single genetic elements promotes their retention, since application of any one selective force co-selects for all the linked resistance genes and for the compound element itself [47,48,60]. Additionally, any gene that can embed itself within a complex xenogenetic element can take advantage of co-selection. This particularly applies to mobile DNA elements, whose presence can both expand the phylogenetic distribution of the parent element, and increase the number of possible interactions with other elements, thus generating enormous complexity through combinatorial assembly [61]. As a consequence, multi-resistance plasmids and other resistance loci have become home to an extraordinary array of insertion elements and transposons [62,63,64].

## 4. History of the Human Microbiota

The human microbiota is a complex ecosystem, consisting of multiple species that interact with each other, and with the host, to generate a dynamic community. The final microbial assemblage at any particular time, and at any one body site, is determined by ecological and evolutionary forces, including: (i) colonization processes, themselves subject to dispersal, ecology and opportunity; (ii) recruitment to particular body sites, driven by interactions between microbial and host genotypes; and (iii) competition between cells for resources, coupled with natural selection [65]. Within such a framework, each individual human can be regarded as an island of habitat, ready to be colonized by dispersing microbiota [12].

Just as humans have had significant effects on the world’s ecosystems, our activities have also had adverse effects on our internal ecosystems, perturbing our microbiota. These perturbations have roughly corresponded with the key transitions that define the Anthropocene: the origin of farming; the industrial revolution; and the “Great Acceleration” since the 1950’s [7,66]. To fully understand the changes that might have occurred over human evolutionary history, it would be useful to have a better idea of what an ancient human microbiota looked like.

There are three possible ways to reconstruct our ancestral microbiota. These are: to examine the microbiota in our closest relatives, the great apes [67]; to examine microbial fossils in human coprolites, fossil dental calculus or other preserved material [68,69]; or to examine the microbiota of extant human cultures that have lifestyles similar to our hunter-gatherer ancestors [25,26]. These three approaches all yield results that are in broad agreement.

Changes to the composition of gut microbiota in the great apes are clock-like, and consistent with the dates of their evolutionary divergence [67]. In contrast, the human microbiota has evolved at an accelerated rate since diverging from chimpanzees, and has lost ancestral diversity. This loss of diversity was accompanied by an increase in taxa associated with diets containing animal products [67]. In this context, it would be interesting to compare the human microbiota with that of baboons, which might have a diet similar to early hominines.

It is also clear that the habitual use of fire for preparing food from about 350,000 years ago [70] may have relaxed the selection for retaining some microbial species, in particular, for taxa capable of dealing with the more refractory components of plant foods. A shift in allocation of resources, from the gut to supporting a larger brain, results in humans having a relatively small gut for our body size [71]. Further, the small intestine accounts for over half the gut volume in humans, while in the other great apes it occupies only 14%–29% [72]. Both these factors mean that there is comparatively less volume for the high microbial population density typical of the large intestine, and this may in turn have lowered overall microbial diversity during human evolution.

In some cases, the microbiota of ancient humans has been preserved as fossils. Given the caveats of bacterial preservation and DNA recovery, these fossils can shed light on ancient microbiota. Coprolites from 8000 to 1400 years B.P. show that early humans had microbiota that was similar to rural, rather than cosmopolitan communities [73]. Calcified dental plaque documents compositional shifts and declining diversity in the oral microbiota during the transition from hunter-gatherers to the Neolithic, and again around the industrial revolution [28].

Studies on human populations with lifestyles similar to our pre-agricultural ancestors confirm these general conclusions. The Hadza of Tanzania [24] and the Amerindian Yanomami [11] both maintain hunter-gatherer lifestyles, and have much higher levels of microbial diversity than other human populations. The Yanomami, in particular, have had little or no outside contact, and represent a human population that may have been isolated for 11,000 years. They have unprecedented diversity of both microbial species and gene functions [11]. The Asaro and Sausi of Papua New Guinea are traditional societies that rely on subsistence agriculture, and also have very high diversity in their microbiota. A significant proportion of their microbial taxa are not found in US populations [74].

In all the traditional populations, there is a high microbial α-diversity (diversity of species within the microbiota of an individual), attributed variously to retention of diverse taxa that deal with refractory foods, frequent meals, food seasonality or increased environmental exposure [11,74]. In comparison, there is less α-diversity in subsistence agriculturalists from Venezuela and Malawi, and even less in Italian and US metropolitan populations [11,24,26,74]. In contrast, microbial β-diversity (differences in species composition between individuals), is much higher in westernized societies [11,74]. Microbial assemblages in westernized populations may be sufficiently different to reliably identify individuals [75].

These data suggest that ancestral humans had high microbial diversity, but individuals carried roughly the same diverse flora. Why then do westernized humans differ so much in their microbial carriage, when there are apparently less species for them to acquire? One explanation is that the greater dietary ranges of individuals in modern societies drive the selection of a greater range of microbial species. Another explanation, often cited in the literature, is that there are decreased dispersal rates amongst westernized populations [74]. On the face of it, improvements in sanitation and hygiene would appear to support this view. However, this does not sit easily with the vastly increased opportunities for exposure to organisms from virtually any continent, brought about by globalization (Figure 1). The rapid spread of infectious disease agents [29,76] and newly emerging antibiotic resistance genes around the world [77,78] demonstrate that dispersal of microorganisms is perhaps more efficient than it has ever been.

We propose an alternate view, that the elevated β-diversity in modern humans is a result of interplay between ecological and evolutionary factors. These include interruption to colonization opportunities, stochastic sampling from a depauperate pool of microbial species, and the sequential imposition of multiple bottlenecks that affect both species and genetic diversity. Under this hypothesis, each individual in modern society is a microbial island, upon which the potent effects of diverse antimicrobial agents are unleashed, at different intervals, and in different relative orders, unique to each person.

What are the effects of antibiotic administration on the human microbiota? Antibiotic therapy leads to rapid shifts in composition, and a decline in microbial diversity. Susceptible microorganisms are depleted while resistant cells increase in relative abundance, with the consequent fixation and persistence of resistance determinants [79]. Although communities do recover after cessation of antibiotic therapy, they may never return to their original diversity, and often still carry the relevant resistance genes, even four years later [80]. In many populations, the average time period between different courses of antibiotics is much less than four years. On any given day, between 1% and 3% of Europeans are on antibiotic therapy [81].

The response of the gut microbiota to antibiotics differs between individuals [82], consequently α-diversity is decreased, while simultaneously increasing β-diversity. The same phenomenon is likely to occur even within species, because resistant clonal lines are favored by antibiotic selection. During antibiotic therapy, declines in intra-species genetic variation have been observed, caused by significant shifts in clonal population structure. These changes persisted over the entire course of several studies (2–4 years), and have been observed in genera as diverse as *Bacteroides*, *Enterococcus* and *Staphylococcus* [79,83,84]. The consequences of repeated antibiotic therapy would then be a series of genetic bottlenecks [85], each of which takes the microbial assemblages of individuals on increasingly divergent paths. High coverage sequencing of metagenomic DNA will be needed to resolve microbial strains within the microbiota of individuals [86].

## 5. Conclusions

The clear conclusion emerging from the literature is that the diversity of the human microbiota has been declining, initially during our divergence from the great apes, but progressively more so during the Neolithic, Industrial Revolution and Modern Eras (Table 1). The reasons for this decline are multifactorial, and involve intersecting ecological and evolutionary drivers. Changes in diet through the use of fire in food preparation, and the invention of agriculture, probably both reduced α-diversity, resulting in a loss of species [87], and a loss of gene functions in microbial populations [11,24].

**Table 1:** A proposed history of ecological and evolutionary effects on the human microbiota.

Timeframe	Ecological or Evolutionary driver	Possible effect on microbiota	Possible effect on microbiome
Pre-350,000 bp ^A^	fire & diet gut *vs.* brain investment	↓ α-diversity	speeds molecular clock
Neolithic 10,000 bp	farming & diet	↓ α-diversity zoonotic exchanges	↓ gene functions
Industrial revolution 18th century	processed foods pollutants, heavy metals globalization	↓ α-diversity selection ↑ dispersal	selection for *mer* operon
Modern era 1930’s	disinfectants sanitation	↓ α-diversity ↑ β-diversity?	selection for *qacE*, spread of class 1 integron
First antibiotic 1930’s	sulphonamides	↓ α-diversity selection	selection for *sul1*
Antibiotic era 1950’s on	diverse antibiotics caesarean section bottle feeding globalization sanitation	↓ α-diversity ↓ transmission ↓ transmission ↑ dispersal ↑ β-diversity	selection for resistance genes ↓ intra-species genetic diversity

A = before present.

However, it is during the last 300 years that the most significant changes are likely to have occurred. Exposure to pollutants and the increasing use of antimicrobial compounds has imposed strong selection directly on all components of the microbiota, in a manner that was unknown in the past. Because of the essentially stochastic nature of antibiotic therapy, and the variation in individual responses [82], this leads to a series of population, species and genetic bottlenecks within the microbiota of individuals [85], potentially leading to unique microbial assemblages in every person. Of course, many other factors might be at play, including the consumption of increasingly hygienic and processed food [23], and reduced transmission between individuals due to sanitation, caesarian section and bottle feeding [7,74,87]. Further, declining α-diversity in each generation reduces the diversity available for transmission to the next generation, and does so in a step-wise manner [87].

It is useful to think of each individual human as an island, colonized and populated by microorganisms. From this perspective, many of the principles of island biogeography become useful and informative. The human body is originally colonized by microorganisms from relatives, and from the environment, and these assemblages undergo progressive changes to reach a final, climax community as an adult [88,89]. In the modern world, declining microbial exposure and decreased α-diversity in our relatives limits the diversity of potential colonizers, and thus makes the eventual climax community less predictable. Each island of microorganisms is also then exposed to different agents of selection, which preferentially remove some elements of the assemblage, and lower α-diversity at both species and genetic levels [83,85,90]. Each island experiences these perturbations at different times, with different agents of selection, and thus each human microbial island has a different ecological and evolutionary history. This sequential series of independent bottlenecks [85,91] then generates the elevated β-diversity that characterizes modern populations [11,74].

Depauperate ecosystems are less resilient than richly diverse ecosystems, and this appears to be true of our internal microbial ecosystems as well. Perturbation to the human microbiota is now being associated with an increasing list of diseases. These include many diseases of uncertain etiology that have rapidly increased in frequency in the last 50 years, such as allergic disorders, chronic inflammatory conditions, and autoimmune diseases [10,92]. The microbiota is also thought to influence behavior, via feedbacks on brain development [93,94], suggesting that microbiome perturbations might contribute to the increasing frequency of anxiety and depression in the developed world [95].

The interactions between the microbiota and host tissues are essential for good health, yet antibiotics alter microbial physiology and gene expression [96], and if administered during a critical window, can have permanent effects on host metabolism [97]. We are only just beginning to understand the complexities of our internal ecosystems, and with some notable exceptions [98], do not have effective therapies for restoring a “healthy” microbiota. In the quest for such solutions, it would be good to remember that while human genetic diversity consists of allelic diversity in the nuclear and mitochondrial genomes, the potential genetic diversity of the microbiome outstrips this by several orders of magnitude. Managing and maintaining microbial diversity may turn out to be a central part of human health into the future.
